# Establishing and Validating a novel Prognostic Model in the Initial Diagnosis of Non-small Cell Lung Cancer with Bone Metastases

**DOI:** 10.7150/jca.95784

**Published:** 2024-07-02

**Authors:** Bin Li, Deying Su, Xiaoyan Wen, Miaomiao Jia, Ning Xue, Shulin Chen, Chaoju Lou

**Affiliations:** 1 Department of Orthopedics, The First Affiliated Hospital of Zhengzhou University, Zhengzhou 450052, P. R. China.; 2 Research Center for Translational Medicine, First Affiliated Hospital, Sun Yat-sen University, Guangzhou 510006, P. R. China.; 3 Department of Central Sterile Supply, Guanghua School of Stomatology, Affiliated Stomatological Hospital, Guangdong Province Key Laboratory of Stomatology, Sun Yat-Sen University, Guangzhou 510055, P. R. China.; 4 Department of Clinical Laboratory, The Affiliated Cancer Hospital of Zhengzhou University& Henan Cancer Hospital, Zhengzhou Key Laboratory of Digestive System Tumor Marker Diagnosis, Zhengzhou 450008, P. R. China.; 5 State Key Laboratory of Oncology in South China, Guangdong Provincial Clinical Research Center for Cancer, Sun Yat-Sen University Cancer Center, Guangzhou 510060, P. R. China.

**Keywords:** NSCLC, bone metastases, LASSO-Cox regression, prognostic model

## Abstract

**Background:** The aim of this research is to establish and validate a prognostic model for predicting prognosis in non-small cell lung cancer (NSCLC) patients with bone metastases.

**Methods:** Overall, 176 NSCLC patients with bone metastases were retrospectively evaluated in the research. We employed the LASSO-Cox regression method to select the candidate indicators for predicting the prognosis among NSCLC patients complicated with bone metastases. We employed the receiver operating characteristic curve (ROC) and the concordance index (C-index) to assess the discriminative ability.

**Results:** Based on the LASSO-Cox regression analysis, 9 candidate indicators were screened to build the prognostic model. The prognostic model had a higher C-index in the training cohort (0.738, 95% CI: 0.680-0.796) and the validation cohort (0.660, 95% CI: 0.566-0.754) than the advanced lung cancer inflammation index (ALI). Furthermore, the AUCs of the 1-, 2-, and 3-year OS predictions for the prognostic model were higher than ALI in both cohorts. Kaplan-Meier curves and the estimated restricted mean survival time (RMST) values showed that the patients in the low-risk subgroup had the lower probabilities of cancer-specific mortality than high-risk subgroup.

**Conclusions:** The prognostic model could provide clinicians with precise information and facilitate individualized treatment for patients with bone metastases.

## Introduction

Lung cancer remains the highest mortality cancer, with approximately 1.8 million deaths in 2020 1. Non-small cell lung cancer (NSCLC) is the dominant type of lung cancer and accounts for approximately 80-85% of all lung cancer cases. The rest are small cell lung cancer (SCLC) 2. The prognosis of NSCLC patients is poor and up to 57.5% of advanced NSCLC patients present bone metastases when the diagnosis is made 3. Bone metastases can result in the development of skeletal-related events, for example, pain, pathological fractures, impaired mobility, nerve root compression, and hypercalcemia, all of which are associated with loss of function and reduced life quality score 4, 5. Although several parameters for predicting the prognosis of lung cancer patients have been developed, the studies focused on the prognosis of advanced NSCLC patients with bone metastases are rare. It remains challenging to precisely predict the prognosis of NSCLC patients with bone metastases. Hence, it is essential to select reliable prognostic factors for better prediction of the outcome of NSCLC patients with bone metastases and to guide optimal therapeutic regimens.

Advanced lung cancer inflammation index (ALI) is an effective prognostic indicator for with NSCLC patients 6. The ALI is based on the patient's weight, height, serum albumin and NLR at diagnosis. It covers anthropometric, nutritional and inflammatory factors and can be a good predictor of lung cancer prognosis 7. However, many studies have indicated that various other factors have predictive value in the prognosis of advanced NSCLC patients, such as performance status and serum biomarkers. Karnofsky Performance Score (KPS) is a commonly used scale to evaluate the patients' performance status. Typically, it is reported by physicians as a summary score. KPS has been used to predict the prognosis in advanced NSCLC patients 8, 9. The traditional bone turnover marker of alkaline phosphatase (ALP) expressed by osteoblasts, which was related to the prognosis of lung cancer patients with bone metastases 10. Moreover, high ALP levels were thought to be linked to poor survival in small cell lung cancer with bone metastases 11. Our previous study results suggest that aspartate aminotransferase (AST) is a valuable marker for prediction of the prognosis of NSCLC 12. However, screening and combining biomarkers into a prognostic system remains a challenge for NSCLC patients with bone metastases.

LASSO-Cox regression is a hierarchical model approach for detecting important variables and predicting survival outcomes 13, 14. Therefore, according to the LASSO-Cox regression analysis, the purpose for our study was to construct and to validate the prognostic model for NSCLC with bone metastases, which were conveniently used for the prognostic prediction.

## Materials and Methods

### Patients

We retrospectively analyzed 176 patients with stage IV NSCLC from Sun Yat-sen University Cancer Center between January 2011 and December 2015. All patients were randomly split into a training cohort (n = 106) and a validation cohort (n = 70). Patients should meet the inclusion criteria for this study: (1) Patients with stage IV primary NSCLC had bone metastasis at diagnosis, as determined on bone scans. (2) Patients who had not undergone antitumor therapy or bisphosphonate therapy within 3 months prior to enrolment. (3) Patients who had complete clinical records and laboratory data. We had professionals who followed up with patients every six months. Follow-up information was obtained by retrieving medical records, clinic or telephone call. Overall survival was defined as the time from diagnosis of NSCLC with bone metastases to the time of cancer special death. All patients were followed up until death or August 2020.

### Laboratory collection and analysis

Relevant clinical data were collected for each enrolled patients as follows: gender, age, body mass index (BMI), smoking status, tumor history, liver metastasis, brain metastasis, number of bone metastasis, tumor site, category, surgery, chemotherapy, radiotherapy, leukocyte count (WBC), neutrophils count (N), lymphocytes count (L), platelet count (PLT), platelet/lymphocyte ratio (PLR), hemoglobin (HGB), C-reactive protein (CRP), neutrophil/lymphocyte ratio (NLR), red blood cell count (RBC), derived neutrophil-to-lymphocyte (dNLR), glucose/lymphocyte (GLR), The nutritional risk index (NRI), lymphocyte/CRP (LCR), activated partial thromboplastin time (APTT), Glucose(GLU), prothrombin time (PT), alanine aminotransferase (ALT), blood urea nitrogen (BUN), thrombin time (TT), uric acid (UA), fibrinogen (Fbg), calcium (Ca), total protein (TP), glutamyl transpeptidase (GGT), albumin (ALB), globulin (GLOB), ALB/GLOB (AGR), aspartate aminotransferase (AST), creatinine (CRE), alkaline phosphatase (ALP), AST/ALT ratio (SLR), cholesterol (CHO), apolipoprotein A (APOA), apolipoprotein B (APOB), triglyceride (TG), lactic dehydrogenase (LDH), LDH/ALP ratio (LAR), high density lipoprotein (HDL), APOA/APOB ratio (ABR), low density lipoprotein (LDL), CRP /ALB ratio (CAR), cystatin C (Cys-C), prognostic nutritional index (PNI), SII, KPS, modified Glasgow Prognostic Score (mGPS). The calculation formula of indicators are as follows: PNI = Alb (g/L) + 5×lymphocyte count×10^9^ /L. NRI calculating formula with the formula: 1.487×serum albumin concentration (g/L) + 41.7×preoperative weight/ideal body weight (kg). Ideal body weight = 22×height (m)^2^. SII = PLR×Neutrophil×10^9^/L. The mGPS scoring system: CRP ≤ 10 mg/L = 0; CRP > 10 mg/L, albumin ≥ 35 g/L = 1; and CRP > 10 mg/L, albumin < 35 g/L = 2.

### Statistical Analysis

We employed SPSS 25.0 (SPSS, Chicago, USA) and R software version 3.6.2 to carry out all statistical analysis. We adopt LASSO-Cox regression to identify candidate indexes and construct a prognostic model. The prognostic ability of ALI and the prognostic risk score was evaluated by the time-dependent receiver operating characteristic curve (TD-ROC), and the concordance index (C-index). Calibration curve was developed to calibrate the nomogram the 1-, 2-, and 3-year overall survival rates, and it reflects the agreement of predicted survival and actual survival. According to the optimal risk score cut-off (“survminer” R package), NSCLC patients with bone metastases were stratified into high- and low-risk subgroups. The Kaplan-Meier plots and the log-rank test were applied to compare the differences in survival time between the two groups. The relationship between our model, PLR, NLR, NRI, SII, CAR, PNI, and ALI model was identified by Pearson's correlation coefficient. P-value of 0.05 or less were considered statistically significant, and all statistical tests were two-tailed.

## Results

### Patient characteristics

In all, 176 NSCLC bone metastasis patients from Sun Yat-sen University Cancer Center were collected in this retrospective study. All patients were randomly split into a training cohort (n = 106) and a validation cohort (n = 70). *Table 1* presents the characteristics and clinicopathological characteristics of NSCLC bone metastasis patients. In the training cohort, there were 35 (33.02%) females and 71(66.98%) males. The mean age of the patients was 56.47 years. 74 (69.81%) patients have more than 3 numbers of bone metastases. Among them, 90 (84.91%) were adenocarcinoma, 10 (9.43%) were squamous cell carcinoma, and 6 (5.66%) were others. The 1-, 2-, and 3-year OS rate were 56.60%, 35.84%, and 18.87% in the training cohort. For validation cohort, there were 27 (38.57%) females and 43 (61.43%) males. The mean age of the patients was 54.81 years. 50 (71.43%) patients have more than 3 number of bone metastasis. Among them, 58 (82.86%) were adenocarcinoma, 9 (12.86%) were squamous cell carcinoma, and 3 (4.28%) were others. The OS rates of 1-, 2-, and 3-year for the validation cohort were 38.57%, 20.00%, and 7.14%, respectively.

### Prognostic model for predicting OS

**Figure 1:**
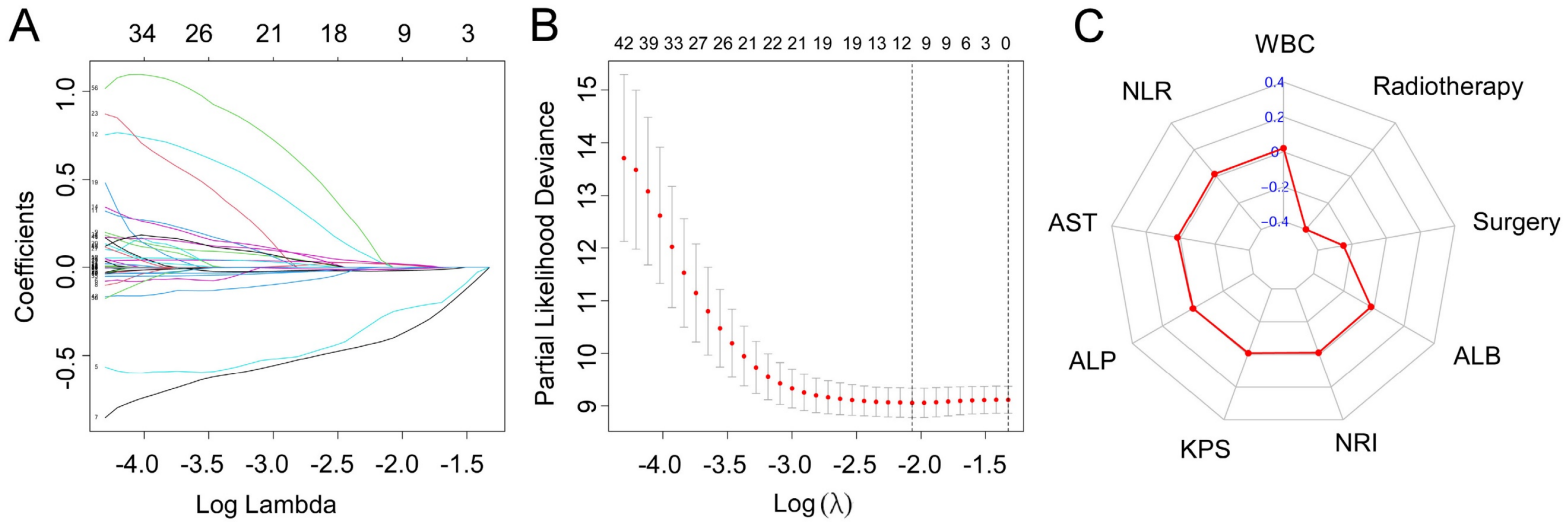
LASSO-Cox regression for potential predictors selection (A). Tenfold cross-validation for prognostic model establishment (B). Radar chart of the indicators in the prognostic model (C).

**Figure 2:**
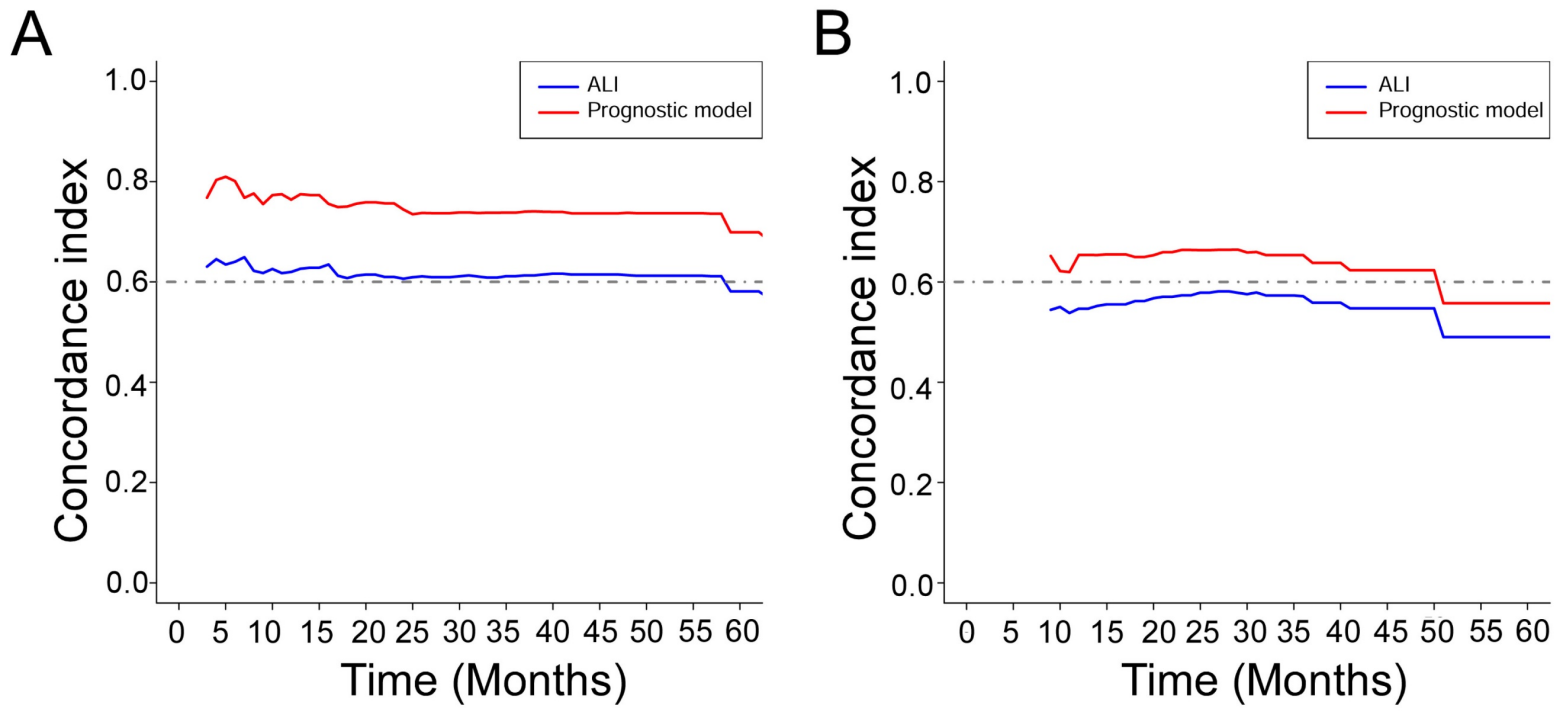
Time-dependent C-index of ALI and prognostic model in the training cohort (A) and the validation cohort (B).

**Figure 3:**
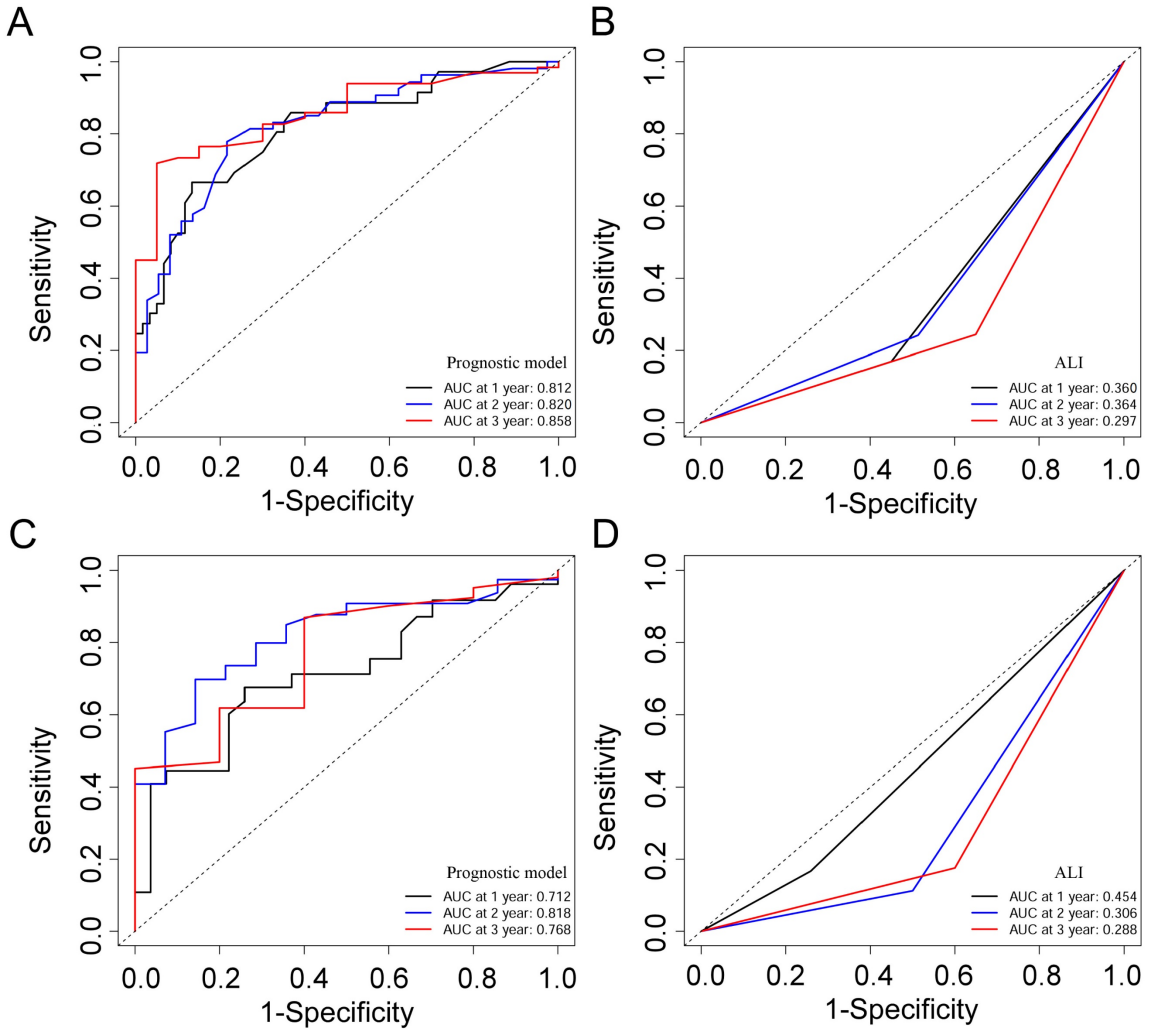
ROC curves of the prognostic model and ALI for 1-, 2-, and 3-year OS in the training cohort (A, B), and the validation cohort (C, D).

Based on the training cohort of OS, we adopted LASSO-Cox regression method to identify candidate index. Analysis of the trajectory changes for each factor are shown in Figure 1A. Model building was performed by tenfold cross-validation. Furthermore, Figure 1B shows the confidence intervals under each λ. The prognostic model was calculated as the following formula: Risk score = -0.2503 * Surgery - 0.3996 * Radiotherapy - 0.0084 * KPS + 0.0212 * WBC - 0.0191 * ALB-0.0003 * ALP + 0.0170 * AST + 0.0174 * NLR - 0.0111 * NRI. Figure 1C shows the detail of these indicators in the prognostic model. We used the C-index to evaluate the predictive ability of the prognostic model and the predictive power of ALI. The C-index of the prognostic model was 0.738 (95% CI 0.680-0.796), which was significantly higher than ALI (0.612; 95% CI 0.560-0.664; *P* < 0.001) in the training cohort. For validation cohort, the C-index of the prognostic model was 0.660 (95% CI 0.566-0.754) and ALI model was 0.568 (95% CI 0.500-0.635) (Table 2). To assess whether the prognostic model was informative beyond ALI model. We performed time-dependent C-index analysis to evaluate the accuracy of these models in the two cohorts (Figure 2A, 2B). As shown in Figure 3A and Figure 3C, the prognostic model AUCs predict the 1-, 2-, and 3-year OS were 0.812, 0.820, 0.858 and 0.712, 0.818, 0.768 in the training and validation cohort, respectively. As shown in Figure 3B and Figure 3D, the ALI model AUCs predict 1-, 2-, and 3-year OS were 0.360, 0.364, 0.297 and 0.454, 0.306, 0.288 in the two cohorts. Significantly, AUC and C-index results showed that our prognostic model had better prediction efficacy compared with ALI model.

### Construction of OS predicting nomogram

**Figure 4:**
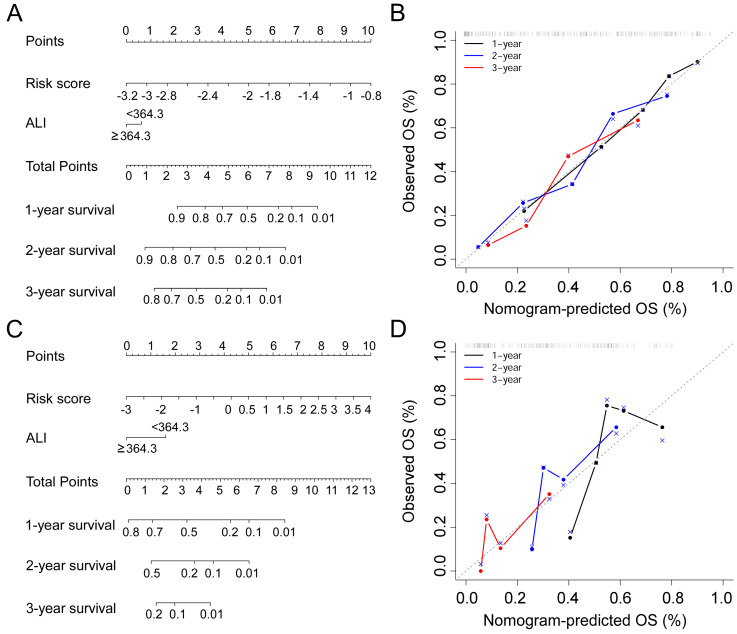
Nomogram for NSCLC patients with bone meta metastasis in the training cohort (A) and the validation cohort (C). Calibration curves for predicting OS in the nomogram in the two cohorts (B, D).

In this study, prognostic risk score = -0.2503 * Surgery - 0.3996 * Radiotherapy - 0.0084 * KPS + 0.0212 * WBC - 0.0191 * ALB - 0.0003 * ALP + 0.0170 * AST + 0.0174 * NLR - 0.0111 * NRI. In order to combine the advantages of the prognostic risk score and ALI, we developed nomograms consisting of the two factors to predict 1-, 2-, and 3-year OS in the training cohort (Figure 4A) and validation cohort (Figure 4C). In the training cohort, the prognostic model (C-index, 0.738 (95% CI 0.680-0.796) and nomogram (C-index, 0.742 (95% CI 0.686-0.797) had similar discrimination ability. In the validation cohort, the C-index of prognostic model was 0.660 (95% CI 0.566-0.754) and the nomogram was 0.663 (95% CI 0.576-0.758). The 1-, 2-, and 3-year survival probability calibration plots showed that the nomogram prediction were well matched with the actual observation (Figure 4B,4D).

### Subgroup analysis according to the risk score

**Figure 5:**
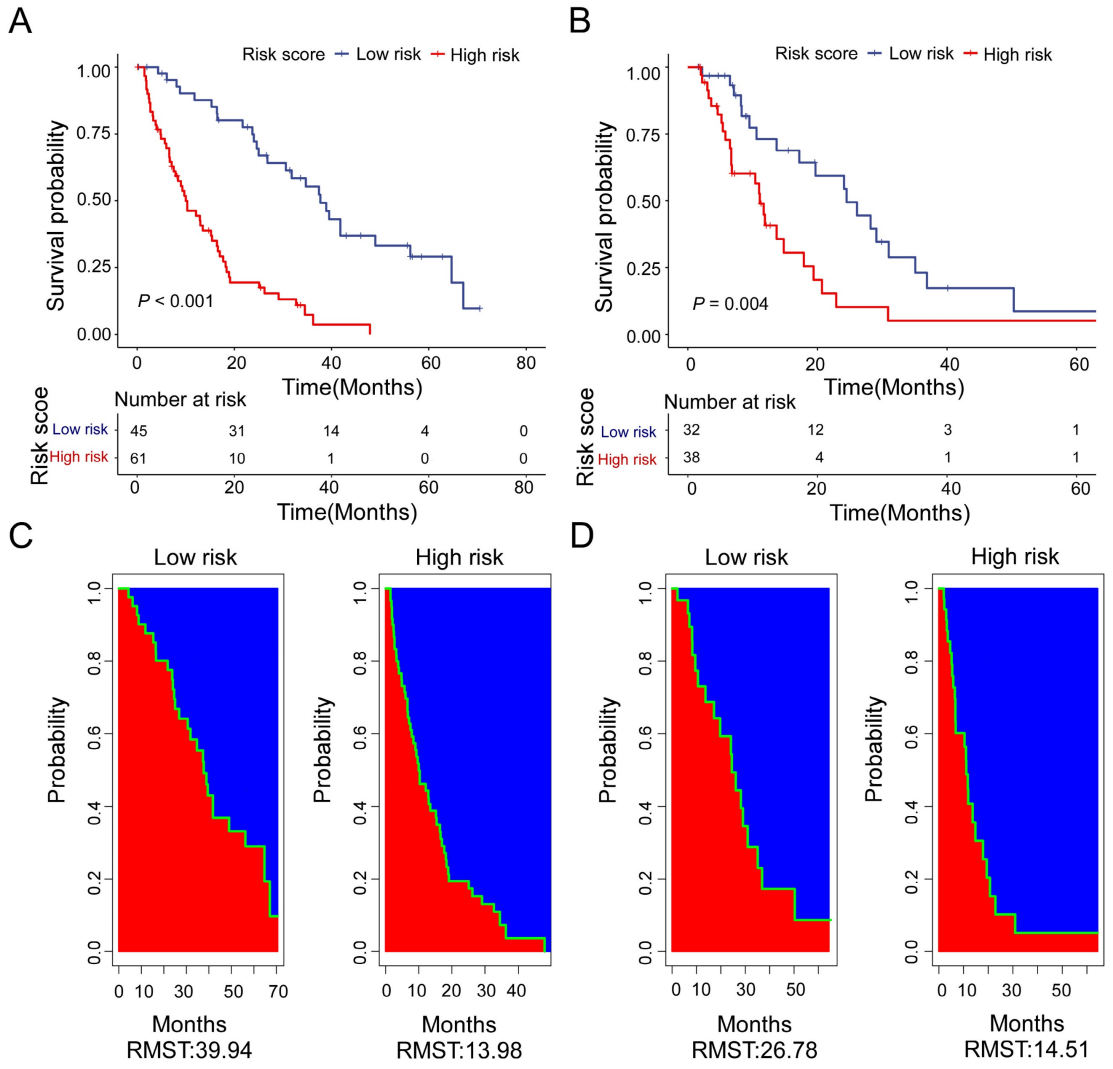
Kaplan-Meier curves for high-risk (red) and low-risk groups (blue) in the training cohort (A) and the validation cohort (B). Estimate of restricted mean survival time (red area) and the restricted mean time lost (blue area) in high-risk group and low-risk group for the training cohort (C) and the validation cohort (D).

Based on the calculation formula of risk score, R package “survminer” and “survival” were used to determine the cut-off values. We adopt the optimal cut-off, the patients with risk score less than -2.18 are in the low-risk subgroup. Meanwhile, the patients with risk score more than or equal to -2.18 are in the high-risk subgroup. There was a significant difference in OS between the two groups. Moreover, the low-risk patients had a better OS benefit than the high-risk patients both in training cohort (Figure 5A) and validation cohort (Figure 5B). For the training cohort, the estimate restricted mean survival time (RMST) values were 39.94 months and 13.98 months for the low-risk subgroup and high-risk subgroup, respectively (Figure 5C). The RTMS of the low-risk subgroup and high-risk subgroup were 26.78 months and 14.51 months in validation cohort (Figure 5D). In all, our results show that the low-risk patients seem to have OS benefit compared with of the high-risk patients.

**Figure 6:**
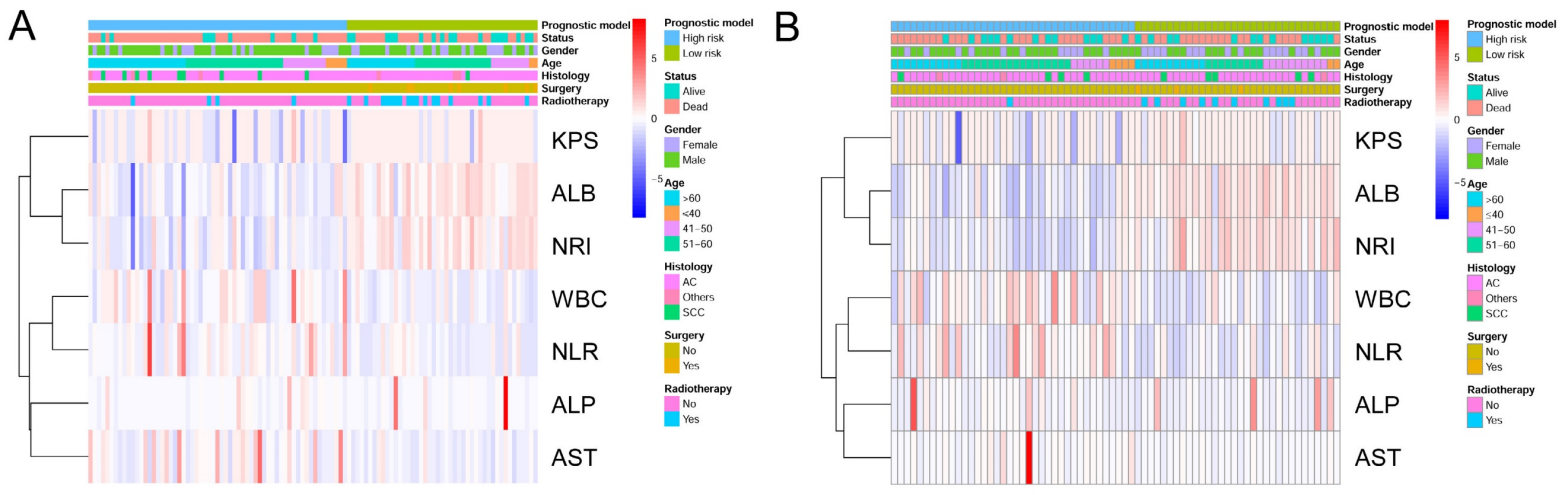
Heatmap was generated by clustering of 9 features across identified NSCLC patients with bone metastasis in the training cohort (A) and the validation cohort (B), respectively.

To identify the high- and low-risk subgroups in the heatmap, unsupervised hierarchical clustering of 9 imaging features (Surgery, Radiotherapy, KPS, ALB, NRI, WBC, NLR, ALP, AST) were performed in the two cohorts (Figure 6A, 6B). The differences in the 9 prognostic variables between the low-risk subgroup and high-risk subgroup are shown in Table 3. In both cohorts, the low-risk subgroup in the KPS, ALB, and NRI levels were significantly higher compare with the high-risk subgroup (*P* < 0.05). Meanwhile, compared to the high-risk subgroup, the low-risk subgroup patients have higher AST (*P* = 0.005), WBC (*P* = 0.002), and NLR (*P* < 0.001) levels in the training cohort. Regretfully, there was no significant difference in serum ALP between high- and low-risk patients in both cohorts (*P* = 0.140,* P* = 0.680).

### The correlation between the prognostic model and other indicators

**Figure 7:**
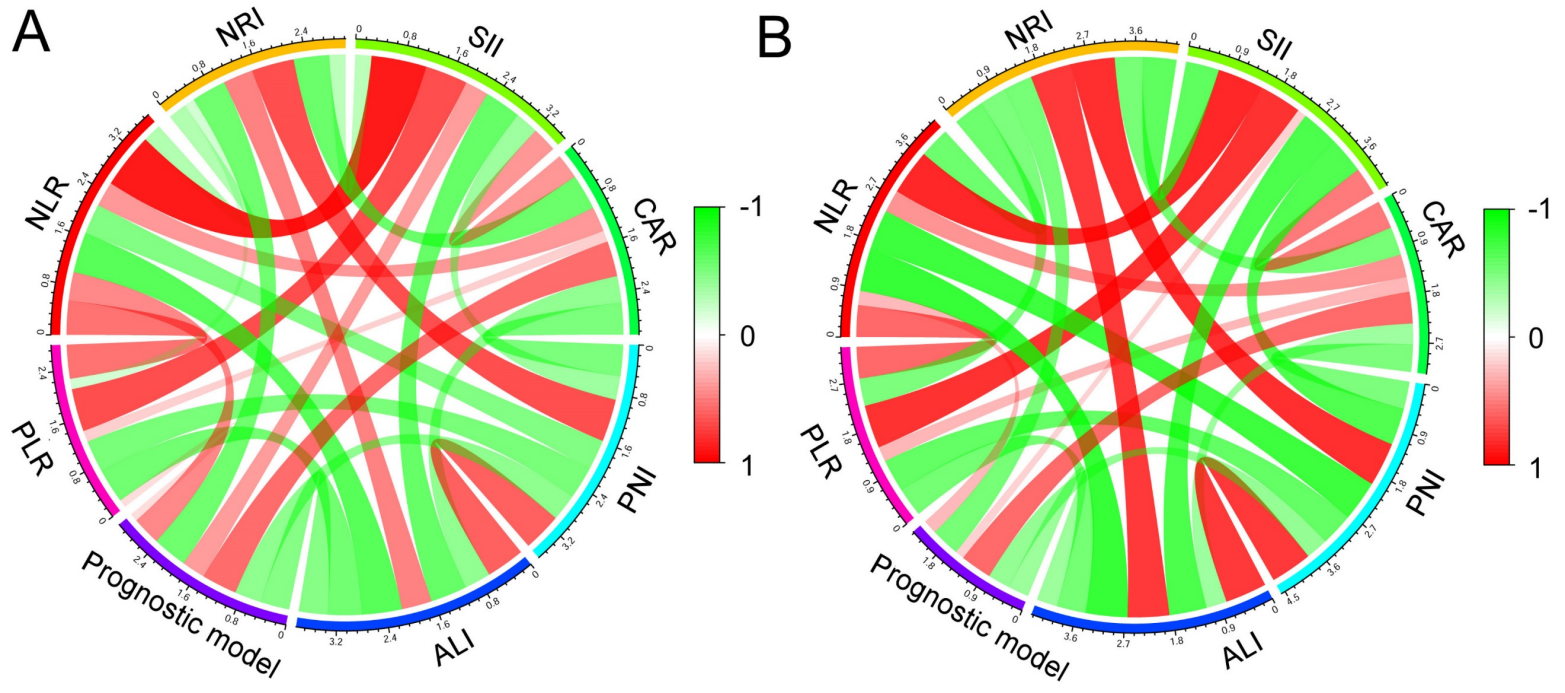
The correlations between the prognostic model, NLR PLR, NLR, NRI, SII, CAR, PNI, and ALI in the training cohort (A) and the validation cohort (B).

The correlations between NLR PLR, NRI, SII, CAR, PNI, ALI, and the prognostic model are shown in Figure 7. Pearson's correlation coefficients (PCC) were used to analyze the correlation between all these indicators. In the two cohorts, the prognostic model and NLR was positively and significantly correlated (PCC: training cohort: 0.462, *P* < 0.001; validation cohort: 0.266, *P* = 0.026). Moreover, similarly with CAR (PCC: training cohort: 0.573, *P* < 0.001; validation cohort: 0.585, *P* < 0.001). In addition, the prognostic model was significantly and negatively correlated with NRI (*P* < 0.001), ALI (*P* < 0.001), and PNI (*P* < 0.001).

## Discussion

In the present research, we employed Lasso-Cox regression method to assess the OS in NSCLC bone metastasis patients and established a multi-parameter prognostic model. Most previous studies used clinical characteristics (gender, age, race, chemotherapy, surgery, radiotherapy, and so on) to construct prognostic model for advanced lung cancer. Besides clinical characteristics, blood biomarkers are widely used in the prognostic analysis of tumors. There are various advantages of blood biomarkers, such as routine detection, easy acquisition, and low cost in primary hospitals. In this study, based on Lasso Cox analysis, our study combined WBC, ALB, ALP, AST, NLR, NRI, KPS with clinical characteristics to construct a novel prognostic model in NSCLC patients with bone metastases for the first time. Our model had superior prediction accuracy and discrimination ability to the ALI model. The prediction model discriminated the NSCLC patients into low- and high-risk groups successfully, with a significant difference in survival probability.

The present prediction model included 9 prognosis-specific factors on the ground of the Lasso Cox regression: radiotherapy, surgery, ALB, NRI, KPS, ALP, AST, NLR and WBC. The possible mechanisms of all these indicators to explain the prognostic values were as follows: ALB is an indicator of nutritional status, and it has many deficiencies 15, 16. Furthermore, many liver-related diseases affect ALB levels 17. Researches have suggested that low serum ALB levels are along with poorer prognosis in breast cancer patients with metastases 18. In addition to serum albumin levels, NRI is a powerful indicator for the evaluation of body nutritional situation. NRI is a useful and independent prognostic parameter for predicting the OS of breast cancer patients and is more accurate than ALB in OS prediction 19, 20. KPS is one of the commonly used scales to evaluate the performance status of patients and it is relevant to the prognosis of NSCLC patients 21. WBCs are peripheral blood indicators widely used to indicate systemic inflammation 22. Leukocytosis could be an independent prognostic parameter for lung and gastric cancers 23. NLR is an important indicator in the prognosis of NSCLC patients. Furthermore, the patients with high NLR have a higher risk of death 24. ALP and AST have been proven to be important predictors of prognosis in NSCLC patients 12, 25. Surgery is the standard treatment for NSCLC patients at operable stage I currently 26. Radiotherapy is also an important modality used for the treatment of lung cancer 27. In this survey, according to LASSO-Cox regression analysis, we integrated all these prognosis related factors (ALB, NRI, KPS, WBC, NLR, ALP, AST, surgery, and radiotherapy) into the prognostic model. Compared with ALI model, this model is more effective in estimating the prognosis of NSCLC bone metastasis patients.

Due to the rareness of reliable prognostic markers for bone metastases, the ALI was originally developed to evaluate the level of systemic inflammation in metastatic NSCLC patients at diagnosis 6. The ALI was developed based on three parameters: NLR, BMI, and serum albumin levels. These factors represent the inflammation-related indicators, the anthropometric indicator and nutrition-related indicators respectively. In a previous study, ALI's prognostic power was higher than many other inflammation/nutrition-based parameters 7. The reason why ALI is better than the other parameters in predicting the prognosis of lung cancer may be that ALI covers anthropometric, nutritional, and inflammatory factors. Therefore, we compared the predictive ability of the model we developed with the ALI. The data showed that the prognostic model had a higher C-index than ALI in the training cohort (0.738 *vs* 0.612, *P* < 0.001) and the validation cohort (0.660 *vs* 0.568, *P* = 0.018). ROC curve analysis showed that our model exhibited better accuracy than ALI in prediction of clinical outcome for 1-year survival (AUC = 0.812), 2-year survival (AUC = 0.820), and 3-year survival (AUC = 0.858) of NSCLC patients with bone metastases in the training cohort. Similarly, the AUC of the prognostic model was higher than that of ALI in the validation cohort. Based on the formulas of the prognostic model risk score, the patients of NSCLC with bone metastases were divided into a high-risk subgroup and a low-risk subgroup. The Kaplan-Meier curves and RMST values revealed that the low-risk group achieved a longer OS than the high-risk group in both two cohorts. All these results revealed that the low-risk subgroup appeared to have an OS benefit compared to the high-risk subgroup.

However, several drawbacks in this research should be taken into consideration. First, this research was a retrospective study at a single-center and it could not rule out all potential biases. An independent data set from another institution is needed to fully verify the prognostic model. Second, the sample size of NSCLC bone metastasis patients is small in both two cohorts. Therefore, in our research, a larger group of patients is needed to validate the prognostic model. Third, this research was only suitable for patients with bone metastasis before any treatment. Finally, the candidate indicators in this study were routine clinical laboratory indicators and other potential indicators were not evaluated, such as miRNAs 28, EGFR 29, circulating tumor cells 30, and serum proteomics 31.

## Conclusion

Based on LASSO-Cox regression analysis, we developed a prognostic model for NSCLC bone metastases including 9 indicators (surgery, radiotherapy, KPS, WBC, ALB, ALP, AST, NLR, and NRI). The prognostic model achieved more accurate prognosis prediction ability than ALI. In this study, the prognostic model provided clinicians with precise information and facilitated individualized treatment for the patients with bone metastases.

## Figures and Tables

**Table 1:** Demographics and clinical characteristics of patients in the development and validation cohort

Characteristic	Training cohort n = (106)	Validation cohort n = (70)	*P* value
	No. (%) or Mean±sd	No. (%) or Mean±sd	
Gender			0.450^a^
Male	71 (66.98%)	43 (61.43%)	
Female	35 (33.02%)	27 (38.57%)	
Age (years)	56.47±9.46	54.81±9.70	0.254^b^
Smoking			0.489^a^
Yes	58 (54.72%)	42 (60.00%)	
No	48 (45.28%)	28 (40.00%)	
Tumor history			0.751^a^
Yes	28 (26.42%)	17 (24.29%)	
No	78 (73.58%)	53 (75.71%)	
BMI (kg/m^2^)			0.445^a^
<18.00	10 (9.43%)	6 (8.57%)	
18.00-24.00	68 (64.15%)	51 (72.86%)	
>24.00	28 (26.42%)	13 (18.57%)	
number of bone metastasis			0.436^a^
1	15 (14.15%)	13 (18.57%)	
2	17 (16.04%)	7 (10.00%)	
≧3	74 (69.81%)	50 (71.43%)	
Liver metastasis			0.178^a^
Yes	21 (19.81%)	20 (28.57%)	
No	85 (80.19%)	50 (71.43%)	
Brain metastasis			0.315^a^
Yes	38 (35.85%)	20 (28.57%)	
No	68 (64.15%)	50 (71.43%)	
Tumor site			0.580^a^
Right	65 (61.32%)	40 (57.14%)	
Left	41 (38.68%)	30 (42.86%)	
Category			0.728^a^
Adenocarcinoma	90 (84.91%)	58 (82.86%)	
Squamous carcinoma	10 (9.43%)	9 (12.86%)	
Other	6 (5.66%)	3 (4.28%)	
Surgery			0.382^a^
Yes	8 (7.55%)	3 (4.29%)	
No	98 (92.45%)	67 (95.71%)	
Chemotherapy			0.364^a^
Yes	93 (87.74%)	58 (82.26%)	
No	13 (12.26%)	12 (17.14%)	
Radiotherapy			0.490^a^
Yes	21 (19.81%)	11 (15.71%)	
No	85 (80.19%)	59 (84.29%)	
WBC (10 ^9^/L)	8.84±4.10	8.65±2.97	0.649^b^
Neutrophils (10 ^9^/L)	6.28±3.81	6.07±2.39	0.578^b^
Lymphocyte (10 ^9^/L)	1.70±0.68	1.66±0.60	0.885^b^
Platelet (10 ^9^/L)	268.05±82.77	288.01±93.11	0.095^b^
HGB (g/L)	133.94±15.93	130.84±16.88	0.345^b^
PLR	179.77±88.71	197.89±107.03	0.201^b^
NLR	4.30±3.43	4.00±1.87	0.448^b^
RBC (10 ^9^/L)	4.56±0.57	4.57±0.56	0.857^b^
dNLR	2.77±2.42	2.49±0.99	0.622^b^
LCR	0.65±1.46	0.63±1.16	0.639^b^
GLR	4.06±3.04	3.89±2.23	0.949^b^
NRI	102.72±9.52	101.18±10.03	0.151^b^
LAR	2.81±3.55	2.42±1.86	0.430^b^
APTT (s)	26.36±4.88	26.52±3.60	0.326^b^
Fbg (g/L)	4.09±1.40	4.51±2.07	0.224^b^
PT (s)	11.14±0.87	11.32±1.01	0.342^b^
TT (s)	17.85±1.42	17.62±2.54	0.716^b^
GLU	5.65±1.71	5.51±1.53	0.869^b^
BUN	5.00±1.51	4.60±1.40	0.080^b^
UA	326.55±93.26	313.41±81.57	0.467^b^
Ca	2.33±0.17	2.33±0.36	0.274^b^
TP (g/L)	71.77±5.53	73.42±5.94	0.089^b^
GGT	50.57±46.14	55.77±72.87	1.000^b^
ALB (g/L)	40.76±4.29	40.06±5.07	0.475^b^
GLOB	31.00±3.87	32.97±6.57	0.031^b^
AGR	1.33±0.23	1.34±0.88	0.093^b^
ALP (U/L)	179.76±314.38	167.37±169.76	0.161^b^
ALT (U/L)	24.67±17.41	25.65±19.69	0.759^b^
AST (U/L)	24.05±10.23	26.67±38.59	0.176^b^
SLR	1.19±0.55	1.13±0.26	0.639^b^
CRE (μmol/L)	68.64±15.10	65.43±17.23	0.303^b^
CRP (mg/L)	19.00±26.10	27.11±37.62	0.654^b^
CHO (mmol/L)	5.11±1.21	4.95±0.97	0.398^b^
APOA (g/L)	1.20±0.22	1.17±0.26	0.386^b^
APOB (g/L)	1.04±0.37	1.00±0.21	0.627^b^
TG	1.39±0.63	1.30±0.70	0.119^b^
LDH (U/L)	325.57±310.49	294.63±202.63	0.804^b^
LDL (mmol/L) HDL (U/L)	3.28±1.04 1.21±0.29	3.20±0.87 1.22±0.50	0.543^b^ 0.680^b^
ABR	1.24±0.36	1.22±0.36	0.759^b^
CAR	0.52±0.77	0.77±1.15	0.652^b^
Cys-C (mg/L)	0.93±0.17	0.94±0.22	0.889^b^
SII	1192.41±1134.48	1202.20±795.42	0.333^b^
KPS	86.51±7.93	86.71±7.21	0.991^b^
mGPS	0.79±0.53	0.91±0.70	0.269^b^
PNI	49.26±5.86	48.37±6.09	0.429^b^

a: Chi-squared test; b: Wilcoxon test. Abbreviations: BMI: body mass index; WBC: white blood cell; HGB: hemoglobin; PLR: platelet/lymphocyte ratio; NLR: neutrophil/lymphocyte ratio; RBC: red blood cell count; LCR: lymphocyte/CRP; GLR: glucose/lymphocyte; NRI: The nutritional risk index; LAR: LDH/ALP ratio; APTT: activated partial thromboplastin time; Fbg: fibrinogen; PT: prothrombin time; TT: thrombin time; GLU: Glucose; BUN: blood urea nitrogen; UA: uric acid; Ca: calcium TP: total protein; GGT: glutamyl transpeptidase; ALB: albumin; GLOB: globulin; ALP: alkaline phosphatase; ALT: alanine aminotransferase; AST: aspartate aminotransferase; SLR: AST/ALT ratio; CRE: creatinine; CRP: C-reactive protein; CHO: cholesterol; APOA: apolipoprotein AI; APOB: apolipoprotein B; TG: triglyceride; LDH: lactic dehydrogenase; LDL: low density lipoprotein; HDL: high density lipoprotein; ABR: APOA/APOB ratio; CAR: CRP/ALB; Cys-C: cystatin C; KPS: Karnofsky Performance Score; mGPS: modified Glasgow Prognostic Score; PNI: prognostic nutritional index.

**Table 2:** The C-index of OS for prognostic model and ALI

Survival prediction	C-index	95 CI%	*P*
Training cohort			
Prognostic model	0.738	0.680 - 0.796	
ALI	0.612	0.560 - 0.664	
Prognostic model vs ALI			<0.001
Validation cohort			
Prognostic model	0.660	0.566 - 0.754	
ALI	0.568	0.500 - 0.635	
Prognostic model vs ALI			0.018

**Table 3:** Distribution of the 9 prognostic variables in the low-risk and high-risk groups

Variable	Training cohort No. (%) or mean ± sd		*P*	Validation cohort No. (%) or mean ± sd		*P*
	Low risk	High risk		Low risk	High risk	
Surgery			0.001^a^			0.054^a^
Yes	8 (17.8%)	0 (0.0%)		3 (9.4%)	0 (0.0%)	
No	37 (82.2%)	61 (100.0%)		29 (90.6%)	38 (100.0%)	
Radiotherapy			<0.001^a^			0.001^a^
Yes	28 (62.2%)	4 (6.6%)		10 (31.3%)	1 (2.6%)	
No	17 (37.8%)	57 (93.4%)		22 (68.8%)	37 (97.4%)	
KPS	88.7 ± 4.6	84.9 ± 9.4	0.015^b^	88.8 ± 4.4	85.0 ± 8.6	0.048^b^
WBC (10^9^/L)	7.5 ± 2.3	9.9 ± 4.8	0.002^b^	7.2 ± 1.9	9.8 ± 3.2	<0.001^b^
ALB (g/L)	43.5 ± 2.8	38.8 ± 4.1	<0.001^b^	44.1 ± 2.7	36.7 ± 4.0	<0.001^b^
ALP (U/L)	210.3 ± 455.2	157.3 ± 140.7	0.140^b^	172.7 ± 173.3	162.9 ± 168.9	0.680^b^
AST (U/L)	20.4 ± 4.8	26.8 ± 12.2	0.005^b^	19.3 ± 6.4	32.9 ± 51.5	0.068^b^
NLR	3.2 ± 1.7	5.1 ± 4.1	<0.001^b^	3.2 ± 1.2	4.7 ± 2.0	<0.001^b^
NRI	108.9 ± 6.8	98.2 ± 8.7	<0.001^b^	108.6 ± 8.2	94.9 ± 6.6	<0.001^b^

a: Chi-square test; b: Wilcoxon test. Abbreviations: KPS: Karnofsky Performance Status; WBC: white blood cell; ALB: albumin; ALP: alkaline phosphatase; AST: aspartate aminotransferase; NLR: neutrophil / lymphocyte ratio; NRI: nutritional risk index.
